# Steel Manufacturing EAF Dust as a Potential Adsorbent for Hydrogen Sulfide Removal

**DOI:** 10.1021/acs.energyfuels.1c04235

**Published:** 2022-03-17

**Authors:** Christian Frilund, Minna Kotilainen, José Barros Lorenzo, Pertti Lintunen, Kimmo Kaunisto

**Affiliations:** † VTT Technical Research Centre of Finland Ltd., P.O. Box 1000, FI-02044 VTT Finland; ‡ Arcelor-Mittal Global R&D, voie Romaine, Maizières lès Metz F-57280, France

## Abstract

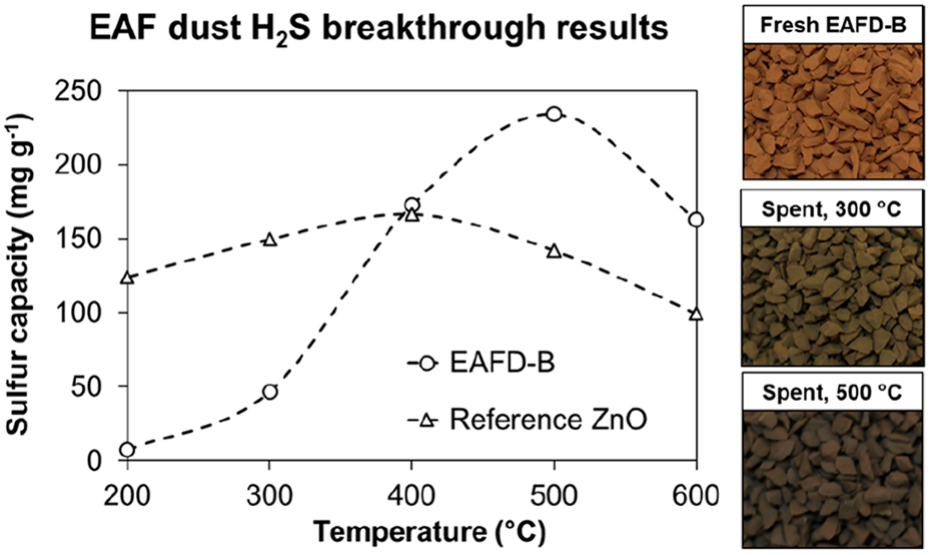

Electric arc furnace dust (EAFD) is a high-volume steel manufacturing byproduct with currently limited value-added applications. EAFD contains metal oxides that can react with H_2_S to form stable sulfides. Hence, the valorization potential of EAFD as an adsorbent material for syngas H_2_S removal was investigated. EAFD from European steel plants was characterized and tested in dynamic H_2_S breakthrough tests and benchmarked against a commercial ZnO-based adsorbent. For this, the EAFD was first processed into adsorbents by simple milling and granulation steps. The EAFD samples exhibited sulfur capture capacities at 400 °C and an SV of 17,000 h^–1^ that correlated with the sample milling times and Zn concentrations. It was verified that only zinc participated in sulfur capture. Yet, both ZnO and the zinc in ZnFe_2_O_4_ were found to be active in sulfidation. At higher temperatures (500 and 600 °C), EAFD sample performance drastically improved and even exceeded the reference zinc oxide performance. The high-zinc (48% by mass) EAFD-B sample exhibited the highest tested performance at 500 °C, with a sulfur capture capacity of 234 mg g^–1^. The results indicate that sufficiently high-zinc-content EAFD could serve as a viable sulfur capture material.

## 1 Introduction

The steel industry is pushed to reduce its environmental impact since it is responsible for approximately 7–9% of total global CO_2_ emissions. As a consequence, the industry is working on transforming the steelmaking technology by two main pathways, the decarbonization of blast-furnace–basic oxygen furnaces (BF–BOFs) and development of innovative direct reduced iron (DRI) electric arc furnaces (EAFs).^1^ The EAF route ensures a lower environmental impact, as it is associated with lower energy demands and recycling potential.^2,3^

The EAF route generates Zn-rich dust as a side stream, especially during the recycling of galvanized steel. Basic oxygen furnace or blast furnace dust also contains zinc but in significantly lower concentrations.^4^ Production of 1 steel ton generates 10–20 kg of fine electric arc furnace dust (EAFD), which is collected by bag filters or electrostatic precipitators.^5,6^ The global generation of EAFD is estimated at about 8 million tons annually.^7^ The EAFD elemental composition varies to a degree, depending on the furnace operating conditions, but it mainly reflects the diversity of the scrap raw materials. Typically, the main elements of the EAFD are iron and zinc, as well as some calcium, chlorine, lead, and small concentrations of several other elements.^8,6^ EAFD is classified as hazardous waste since it contains leachable heavy metals such as lead or cadmium and thus cannot be disposed of in ordinary landfill sites without further treatment, which comprises solidification or stabilization techniques.^9−11^ Consequently, the disposal of EAFD waste has become a significant problem in recent years.

There is wide industrial usage of metal oxides as H_2_S removal adsorbents from industrial gases such as synthesis gas, coke gas from steel production, fuel cell hydrogen, sulfur recovery unit tail gas, and bio/natural gas.^12−16^ In several gas-phase processes, hydrogen sulfide (H_2_S) must be removed to very low concentrations to prevent downstream issues such as catalyst poisoning and fuel cell degradation.^12,17,18^ Although iron oxide^19−21^ is capable of removing H_2_S, zinc oxide is one of the best metal oxides for this purpose.^15,22^ ZnO removes H_2_S by forming a stable sulfide at medium to high temperatures (100–450 °C).^23,24,22^ Other porous materials for H_2_S removal include activated carbons^25^, zeolites^26^, and metal–organic frameworks (MOFs)^27,28^.

**Figure 1:**
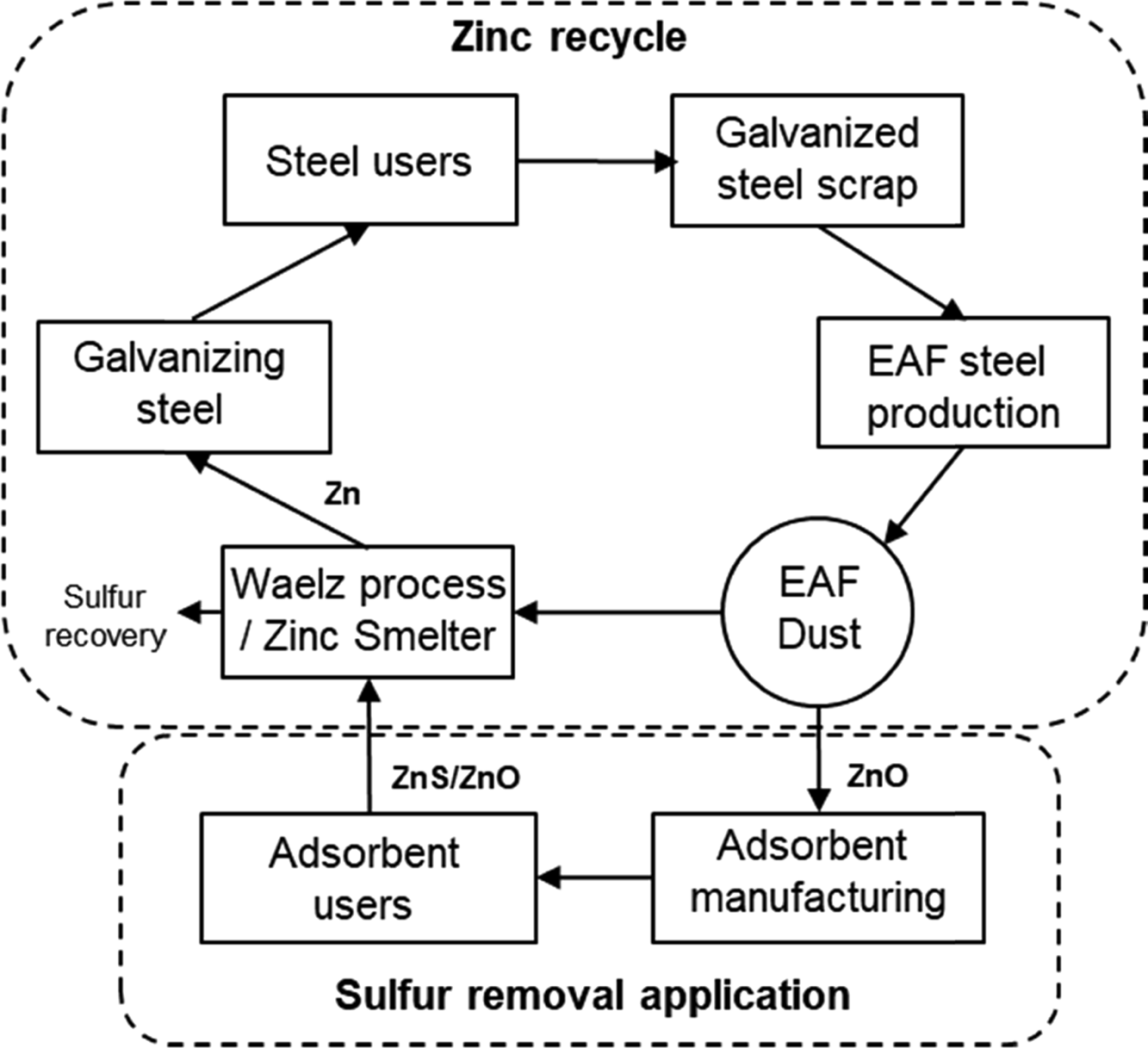
Diagram of a state-of-the-art zinc recycling process with a widening of the material loop by a value-added sulfur removal process.

Primary zinc adsorbent cost is heavily dependent on the ZnO market price, and therefore its use in large-scale gas purification applications may not be economically viable, especially since its regeneration can be challenging.^20,29^ Concurrently, large amounts of zinc-containing side streams are generated in the steel industry. EAFD recycling mainly aims at recovering the zinc portion of the material.^30,6^ Recently, low-end applications for EAF dust have been developed, such as an additive material in asphalt or concrete.^31,4^ The current best option for recycling EAF dust in the zinc raw material loop is by the Waelz process, in which approximately half of the world’s EAF dust is processed.^32,33^ By introducing new applications for the zinc-containing side streams, recycling would become more profitable. One of these applications could be the use of EAF dust for H_2_S removal. Figure 1 illustrates the present state-of-the-art zinc recycling extended with a proposed gas cleaning application.

By substituting primary ZnO adsorbents with the steel industry’s low-cost EAF dust side stream material, the adsorbent price could potentially be reduced, assuming that the adsorbent manufacturing costs are moderate. Also, spent metal oxide adsorbent recycling is potentially streamlined by feeding the adsorbent back to existing EAF dust handling processes. In this study, EAF dust was applied as the adsorbent raw material for lab-scale experiments in sulfur removal from industrial gases. The adsorbent was subjected to extended-duration H_2_S breakthrough tests to determine its applicability for desulfurization.

## 2 Materials and Methods

### 2.1 Raw Materials

Three electric arc furnace dust samples from steel industry side streams were obtained from manufacturers ArcelorMittal (France) and Höganäs (Sweden). EAFD is formed from the volatilization of particles at furnace hot spots like the arc. A steel bath, especially through carbon monoxide bubble bursting, also contributes to dust formation, in which species are transported in the vapor phase to the gas extraction system^34^. In this study, the ArcelorMittal wet dedusting-derived sample is called EAFD-A1, dry dedusting-derived sample, EAFD-A2, and a sample from Höganäs, EAFD-B. As a reference, the experiments featured a commercial ZnO adsorbent (with Al_2_O_3_ additive) of the type ActiSorb S2 manufactured by Clariant, hereafter called ZNO-1.

#### 2.1.1 Material Processing

EAF dust samples were processed into binder- and additive-free granulates for the packed-bed desulfurization tests. First, agglomerates above 0.5 mm were sieved off from the EAF dust and the material was mixed with water to reach a 30% solid content by weight. A 5 kg mixture was processed by continuous wet milling using a Hosokawa Alpine bead mill 90 AHM into a homogeneous dispersion. The standard milling time for the dust was 2 h. Additionally, samples were taken every 30 min during milling for characterization and desulfurization testing.

After the wet milling stage, the dispersions were cast into a flat container and dried in a heating chamber at 80 °C. The milled and dried EAF material was crushed and sieved into a particle size of 1.0–1.25 mm. The granulates were then heat-treated in an air atmosphere corresponding to the sulfur adsorption test temperatures of at least 400 °C and a maximum of 600 °C.

### 2.2 Material Characterization

X-ray diffraction (XRD, Bruker D2 Phaser) was applied to qualitatively determine the main mineralogical phases. Due to the heterogeneity of the EAFD-A1 and -A2 dust samples, the results are reported as mean with the standard deviation (from three analysis samples). The quantitative analysis of zinc comprised wet chemistry precipitative phase separation and inductively coupled plasma optical emission spectrometry (ICP-OES, Spectroblue by AMETEK) analysis of ZnO and other zinc phases along with the total zinc content. Other major elements, Fe, Si, Ca, Al, Mg, and Mn, have been prepared by wet chemistry techniques and quantitatively characterized by X-ray fluorescence (XRF). Quantitative iron analysis was conducted for the total iron content and its specification as Fe^2+^, Fe^3+^, and metallic iron (Fe°). The total carbon content and sulfur content were measured by combustion analysis and IR measurement of the evolved gases (LECO RC612). Karl Fischer titration (Mettler Toledo T7) was applied for water analysis after combustion and a gas volumetry method was applied for CO_2_ determination. The alkali content was determined by inductively coupled plasma mass spectrometry (ICP-MS, Agilent 7700), and the halogens by titration. Minor element compositions were determined by ICP-OES or ICP-MS.

Additionally, samples were characterized with respect to the microstructure, crystal size, specific surface area, pore volume, and thermal behavior. Microstructural and morphological analyses were performed using a Zeiss ULTRA plus (Carl Zeiss) field-emission scanning electron microscope (FESEM) equipped with an energy-dispersive spectrometer (INCA Energy 350 with INCAx-act silicon drift detector, Oxford Instruments). Secondary electron (SE2) and back-scattering (AsB) detectors were used. Thermogravimetric analysis (TGA) was performed using a Netzsch STA 449 F1 Jupiter unit. Approximately 10 mg of samples was analyzed in N_2_ with a heating rate of 10 °C min^–1^ from 40–1000 °C with no holding times. Additional phase analyses of the milled samples were carried out using a PANalytical B.V Empyrean X-ray diffractometer with a Cu Kα radiation source and analyzed using HighScore Plus software with the ICDD database. The crystal sizes of the analyzed phases were calculated using the Scherrer equation in HighScore plus software.

Sample Brunauer–Emmett–Teller surface areas (BET SA) and pore volumes (BJH determined cumulative volume for 1.7–300 nm diameter pores) were measured at −196 °C with N_2_ using a Micrometrics 3Flex analyzer.

### 2.3 Adsorption Tests

**Figure 2:**
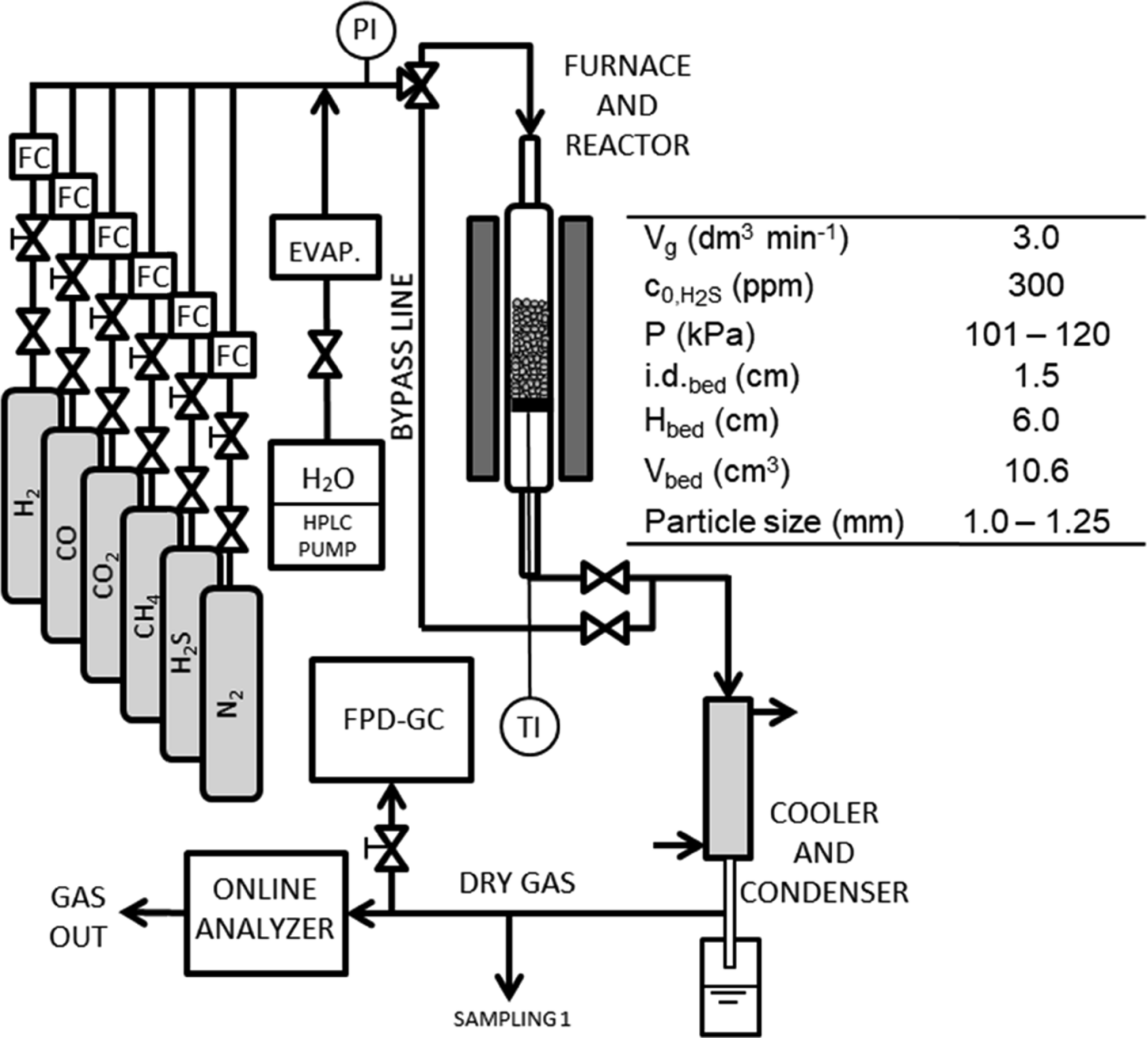
Schematic of the lab-scale adsorption setup and the fixed test conditions.

The adsorption tests involved H_2_S breakthrough tests in a fixed-bed reactor with realistic model gases. Potential adsorption-related issues, such as side reactions, granulate sintering, and agglomeration, were also evaluated. The inner diameter of the quartz reactor was 1.5 cm, and the bed height was fixed at 6 cm. The gases were dosed using Bronkhorst mass flow controllers and water fed with a high-performance liquid chromatography (HPLC) pump to an evaporator. The bottled gases were mixed with the vaporized water in a heated inlet line. The effluent gas was cooled in a condenser tube with a cooling water jacket, after which dried gas analysis was performed. Figure 2 illustrates the experimental setup.

All gas volumes are expressed at standard conditions (273.15 K and 101.325 kPa), and small gas concentrations are reported as parts per million (ppm) in volume terms. The experiments were conducted with a wet gas flow rate, V̇g, of 3 dm^3^ min^–1^ and an H_2_S wet gas concentration of 300 ppm. The nominal desulfurization gas space velocity was thus 17,000 h^–1^. The primary gas represented a typical biomass-based fluidized bed gasification syngas with a volume-based wet composition of H_2_ 35.9%, CO 18%, CO_2_ 12.8%, and 27.3% H_2_O with balance N_2_. Another gas mixture, model biogas, was composed of CH_4_ 53.2%, CO_2_ 35.9%, 5.0% H_2_O, and balance N_2_. For gaseous sulfur species detection, an Agilent 7890A gas chromatograph with a flame photometric detector (FPD-GC) and a GS-GASPRO 30 m long 0.32 mm i.d. column, with carrier gas He, was used. The GC was calibrated using a H_2_S and carbonyl sulfide (COS)-containing gas with concentrations of 200 and 20.1 cm^3^ m^–3^, respectively, and a relative error of ±2%. The GC detection range for H_2_S was estimated at 0.5–150 ppm.

The adsorption breakthrough time was determined using a dry gas breakthrough concentration of 7 ppm (1.75% of the inlet feed concentration). The sulfur adsorption capacity, *S*_cap_, is given on a unit mass basis (mg g^–1^) for a fixed-volume sample at the breakthrough time. It was calculated by integrating the area above the breakthrough curve for the given inlet H_2_S concentration. Effluent H_2_S concentration is reported in the dry gas.

## 3 Results and Discussion

### 3.1 EAF Dust Characterization

#### 3.1.1 Mineralogy

The X-ray diffraction analysis of EAF dust samples was applied for qualitative characterization of the main mineralogical phases, and the diffractograms of EAFD-A1, -A2, and -B are given in the Supporting Information. The XRD results indicated the presence of most of the main elements as oxides or complex oxides of which the most abundant were ZnO, Fe_2_O_3_, and ZnFe_2_O_4_. The diffractogram of EAFD-B shows an intense signal for ZnO, and most of the iron is associated with zinc as zinc ferrite. The EAFD-A1 and -A2 samples demonstrate a higher relative share of zinc as zinc ferrite. Calcium is present as CaO and CaCO_3_, and chlorine as NaCl or PbOHCl.

#### 3.1.2 Chemical Composition

Table 1 presents the results of quantitative chemical characterization of the EAFD samples. In addition to Fe and Zn, the other major elements comprise Ca, Al, Si, Mg, and Mn, which given the EAF formation conditions, are primarily present as simple oxides.

**Table 1:** Main Element Compositions of Fresh EAFD Samples

	**EAFD**		
	**-A1**	**-A2**	**-B**
**Iron (% by Mass)**			
Fe^3+^	35.9 ± 0.6	32.6 ± 0.8	24.4
Fe total	39.2 ± 0.6	35.7 ± 1.0	26.5
**Zinc (% by Mass)**			
Zn as ZnO	12.0 ± 0.7	13.8 ± 1.3	41.1
Zn as zinc ferrite	9.8 ± 0.8	8.9 ± 0.9	6.9
Zn total	19.7 ± 3.2	22.6 ± 1.6	48.1
**Others (% by Mass)**			
Si	2.7 ± 0.2	2.8 ± 0.4	0.8
Ca	5.8 ± 0.4	7.6 ± 0.5	2.3
Al	0.5 ± 0.01	0.6 ± 0.1	<0.5
Mg	0.01 ± 0	0.02 ± 0	<0.5
Mn	0.03 ± 0	0.03 ± 0	1.2

Insignificant amounts of metallic zinc and iron are observed in the samples, which are consistent with the oxidizing atmosphere in the electric arc furnace. The EAFD-A samples exhibit a zinc concentration of 19.7–22.6% by mass, though with a significant standard deviation. Based on the XRD results, the other zinc phases are identified as zinc ferrite. The EAFD-B sample is considerably richer in zinc oxide, while the zinc ferrite concentration is smaller. The EAFD-B total zinc composition stands at 48.1% by mass, which is twice as much as in the EAFD-A samples. The wet dedusting sample, EAFD-A1, and the dry dedusting sample, EAFD-A2, had similar zinc contents. The ratio of zinc in ZnO over ZnFe_2_O_4_ is approximately 0.5–0.6.

Due to the high zinc concentration, the EAFD-B iron composition is under 30%, while for EAFD-A samples, it ranges from 35 to 40%. Ferric iron (Fe^3+^) may exist in several complex oxide forms, including Fe_2_O_3_, Fe_3_O_4_, and zinc ferrite. Ferrous iron (Fe^2+^) can exist in the FeO form or be bound in Fe_3_O_4_. Most of the iron in the EAFD samples is in the Fe^3+^ oxidation state and is likely combined with zinc to form ZnFe_2_O_4_. EAFD-A samples exhibit significantly higher concentrations of calcium and silica than EAFD-B. EAFD-B holds 1.2% by mass manganese, while the EAFD-A samples are almost manganese-free.

Iron oxides stem from the steelmaking ferrous burden, while basic oxides such as CaO and MgO evolve from the fluxes involved in the fabrication. Other oxides, such as SiO_2_ and Al_2_O_3_, originate from the nonferrous part of the raw materials. Volatile elements like Zn and Pb as well as other trace elements also originate from the scrap raw material load. Table 2 presents other element compositions.

**Table 2:** Analysis Results of Other Elements in the Fresh EAFD Samples

	**EAFD**		
	**-A1**	**-A2**	**-B**
**Nonmetals (% by Mass)**			
Cl	3.6 ± 0.6	2.9 ± 0.2	0.3
F	0.2 ± 0.1	0.2 ± 0.1	0.03
C as CO_2_	0.3 ± 0.1	0.6 ± 0.3	0.3
total C	2.8 ± 0.1	3.0 ± 0.2	0.5
S	0.5 ± 0.1	0.4 ± 0.1	0.1
H_2_O	1.3 ± 0.1	1.8 ± 0.2	0.7
**Alkalies (% by Mass)**			
K	1.0 ± 0.2	0.9 ± 0.1	0.9
Na	1.5 ± 0.2	1.1 ± 0.2	0.7
**Other Metals (% by Mass)**			
Pb	1.3 ± 0.1	1.8 ± 0.2	0.1
Cu	0.3 ± 0.1	0.3 ± 0.1	0.03
Cr	0.7 ± 0.1	0.6 ± 0.1	0.06

The results indicate that there is a higher concentration of carbon in the EAFD-A samples than in EAFD-B. Calcium and magnesium can be found as carbonates. However, most of the carbon in the samples was not bound as CO_2_ but other forms (e.g. coke). The total sulfur concentration exceeds that of zinc sulfide, indicating the presence of other sulfur phases. The water concentration varies between 0.7 and 2.2% by mass. For halogens, alkali metals, and other metals, it can be observed that EAFD-B contains significantly smaller quantities of all of these elements compared to EAFD-A. A full analysis of trace elements is available in the Supporting Information.

Thermogravimetry tests in N_2_ show that the EAFD samples exhibit sufficient thermal stability at the applicable desulfurization temperature range. The TGA diagrams are available in the Supporting Information.

### 3.2 Sulfur Removal

**Figure 3:**
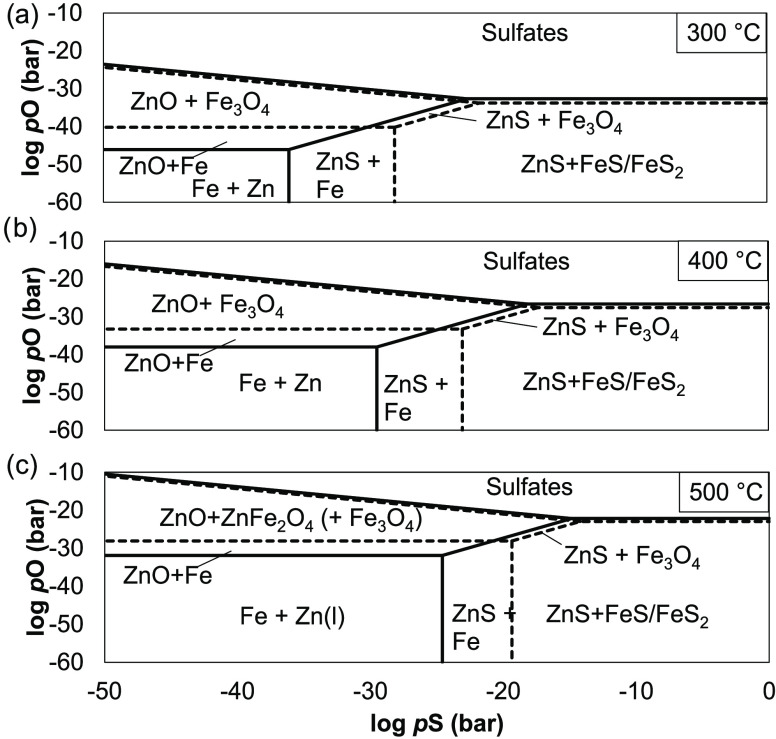
Phase stability diagram of a Zn–Fe–O–S system at: (a) 300 °C, (b) 400 °C, and (c) 500 °C. Zn-boundaries are shown with solid lines and Fe-boundaries with dotted lines. The *x*-axis depicts the sulfur partial pressure and the *y*-axis depicts the oxygen partial pressure. The data was retrieved from HSC Chemistry 8 Zn–O–S/Fe–O–S calculations in addition to a Factsage 8.1 derived Zn–Fe–O–S system.

The characterization results show that the EAFD samples are iron–zinc mixtures, with the presence of other metal oxides and smaller amounts of nonmetals. Both iron oxides and metallic iron show activity for the sulfidation reaction at suitable conditions. To investigate their thermodynamic potential for sulfidation, Figure 3 presents phase stability diagrams that feature simplified depictions of a Zn–Fe–O–S system.

Figure 3 illustrates that in an oxidative atmosphere, sulfates are the predominant stable species, although their share diminishes with increasing temperatures. ZnS exists as a stable species at lower sulfur partial pressures than FeS, and both favor lower temperatures. The main sulfidation reactions for zinc and iron oxides are generalized as
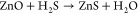
1
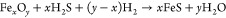
2
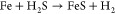
3These reactions can be considered nearly irreversible due to the favorable thermodynamics leading to low H_2_S partial pressures at the solid surface. The intrinsic kinetics of sulfidation of iron oxides is slower than with metallic iron, while metallic iron sulfidation is slower than for ZnO. Calcium oxide, a minor component present in EAFD, is also able to react to form a stable sulfide, though it suffers from poor kinetics and is therefore relevant only at high temperatures (above 600 °C).^35,36^ Mixed metal oxides, such as zinc ferrites and zinc titanates, have previously been investigated in an effort to combine the beneficial properties of multiple oxides to prevent reduction and improve dispersion and porosity.^37−39^ Zinc ferrite is affordable, has an excellent sulfur capture capacity, and exhibits decent regeneration properties. It is still limited to temperatures below 600 °C due to the reduction of zinc.^39,40^ Zinc titanate features an equally good sulfur capture rate and a slower reduction rate. It is generally applied at high temperatures, which limits removal to residual H_2_S concentrations of >10 ppm.^41,37,42^

From Figure 3 it can also be observed that at moderately reducing conditions, iron in metallic form is more stable than metallic zinc. With the presence of H_2_ or CO, reduction of iron oxide proceeds according to
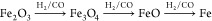
4At elevated temperatures, iron exists in multiple phases, depending on the reductive potential of the atmosphere. A reduction to FeO or metallic iron may also promote the formation of iron carbide, Fe_3_C/Fe_2_C, which reduces the sulfidation capacity and can negatively affect the mechanical strength of the adsorbent.^39^ Furthermore, metallic iron may catalyze the decomposition of CO to form solid carbon.^43^ Although thermodynamic analysis suggests that iron carbides may form at reducing syngas conditions at mid- to high temperatures, Ayala et al.^39^ experimentally verified that no carbon formation occurs in coal syngas for iron oxide samples. Additives such as silicon dioxide and sodium carbonate can also be effective at inhibiting soot formation.^40^

#### 3.2.1 Relative H_2_S Breakthrough Performance

**Figure 4:**
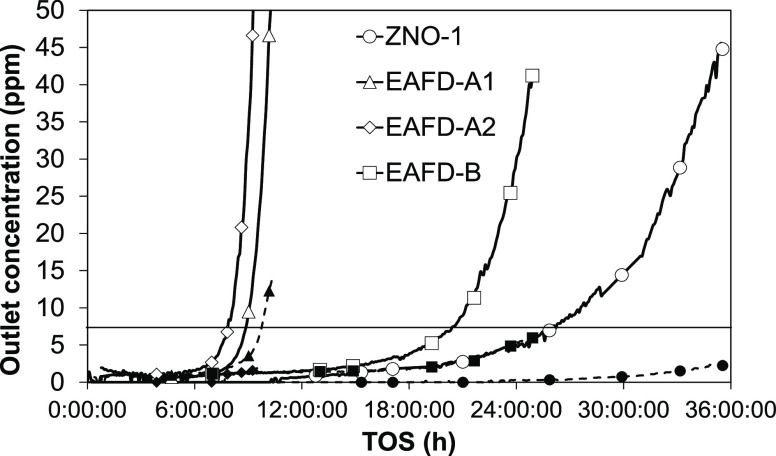
Breakthrough curves of H_2_S and COS in 400 °C syngas. White symbols represent H_2_S and black symbols, COS. The solid horizontal line depicts the breakthrough concentration.

The relative performance of adsorbents was determined in syngas at 400 °C. Since the sulfur capture efficiency is compromised if COS formation occurs, both H_2_S and COS breakthrough curves are presented in Figure 4. The sample packed densities are available in Table 3.

**Table 3:** Spent 400 °C Sample Analysis Results^a^

		**EAFD**		
	**ZNO-1**	**-A1**	**-A2**	**-B**
***S*_cap_** (mg g^–1^)	166 ± 7.5^b^	73	64	170
**ρ** (g cm^–3^)	1.14 ± 0.04	0.95	0.85	0.90
**Zinc (% by Mass)**				
Zn as ZnS	31.8	11.9 [0.01]	12.1 [0.01]	29.2 [0.07]
Zn as ZnO	31.7	1.4 [12.0]	1.7 [13.8]	10.1 [41.1]
Zn as zinc ferrite	0.8	6.8 [9.8]	6.4 [8.9]	3.6 [6.9]
Zn°	2.6	0.5 [0.04]	0.4 [0.03]	2.01 [0.06]
**Iron (% by Mass)**				
Fe^3+^		39.6 [35.9]	37.0 [32.6]	24.1 [24.4]
Fe^2+^		2.0 [2.1]	2.5 [1.7]	2.0 [0.84]
Fe°		1.2 [1.2]	3.3 [1.3]	4.0 [1.2]
**Other analysis**				
BET SA (m^2^ g^–1^)	22.5 [42.7]	11.9 [15.8]	14.1 [18.6]	13.0 [16.0]
Pore V (cm^3^ g^–1^)	0.16 [0.24]	0.07 [0.09]	0.08 [0.10]	0.08 [0.09]

a Fresh sample results are indicated in brackets.

b Repeated four times.

EAFD-B, with the highest Zn-content of the EAFD samples, exhibited around 15% shorter breakthrough time than ZNO-1. Due to the sample density differences, the capture capacities were equal on a mass basis. EAFD-B contained over 30% less zinc than ZNO-1, yet achieved similar performance. However, the tested EAFD samples contain no binder or additives and therefore principally differ from the reference sample ZnO-1. The relative standard deviation of the ZNO-1 capture capacity was 4.5%, which represents the total error of the experimental setup. The H_2_S removal performance of the EAFD-A1 and EAFD-A2 samples was significantly weaker, evidently due to the lower Zn-content. Table 3 shows the spent sample iron and zinc characterization from the 400 °C syngas runs that were operated to partial saturation (*C*/*C*_0_ of 0.1–0.3).

The characterization results illustrate that both ZnO and the zinc present in ZnFe_2_O_4_ are reactive. This is similar to previous findings, where consumption of the zinc portion of franklinite was demonstrated, and both ZnO and ZnFe_2_O_4_ were verified zinc sources for ZnS formation^38^. The BET surface area and pore volume decrease are consistent with the extent of sulfidation that occurred for the tested samples, with the larger S^2–^ occupying a larger volume than O^2–^. From the analysis, the share of the total zinc that is sulfided for EAFD samples amounts to between 55 and 65%, while for ZNO-1, it is approximately 50%. Part of the zinc is reduced to the metallic form, in particular for sample EAFD-B. Similar behavior was observed with sample ZnO-1. For all of the EAFD samples, ZnO is more readily converted, in contrast to ZnFe_2_O_4_. The iron characterization results show that there is no significant increase in Fe^2+^ concentration, which indicates little to no FeS formation. XRD analysis of the spent samples confirms that no FeS is formed. Thus, only the zinc species contribute to H_2_S removal at the test conditions.

In sulfur-rich syngas, an H_2_S-COS equilibrium is formed.^44,45^ An empty bed test was performed to determine the extent of COS formation in the system at 400 °C. The test yielded a 5 ppm COS concentration at the outlet. As Figure 4 indicates, COS breakthrough with all EAFD samples is moderate or it was not observed before the H_2_S breakthrough had occurred. At the H_2_S breakthrough, the COS/H_2_S ratio is in the range of 0.1–0.2 for the EAFD samples. The ZNO-1 run gives a lower ratio, indicating superior COS control, likely due to the higher ZnO concentration in the sample. Mitigating issues related to COS formation comprises the addition of metal oxides such as TiO_2_ or Al_2_O_3_ to the adsorbent material, which are active in the hydrolysis of COS^46^.

#### 3.2.2 Milling Time

**Figure 5:**
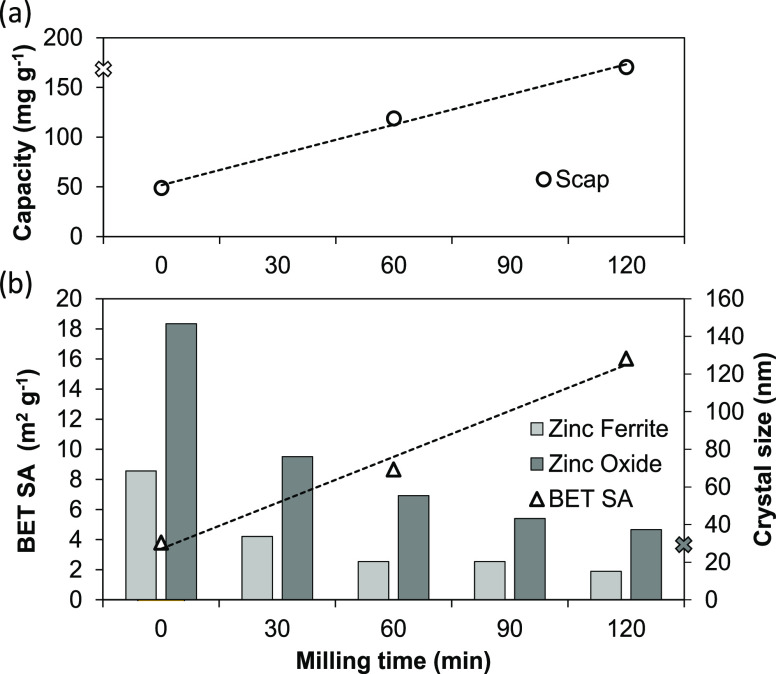
Effect of EAFD-B milling time on (a) sulfur capture capacity in 400 °C syngas (linear trend line R2 is 0.99). (b) BET SA (linear trend line R2 is 0.98) and the mean crystal size (ZnO and ZnFe_2_O_4_). The ×-symbol represents reference ZNO-1 results.

Milling is a simple, yet effective, method to influence powder material morphology, crystal structure, particle size, and porosity.^47^ It is therefore identified as a possible processing step for the production of high-performance EAFD-based adsorbents. Figure 5 presents the sulfur capture capacity in a syngas atmosphere at 400 °C as a function of milling time, along with the crystal size and BET surface area results of sample EAFD-B.

The average ZnO crystal size was reduced from 147 to 37 nm during the 2 h milling period, with the size almost halving in the first 30 min. The 2 h milled EAFD-B crystal size is comparable to the commercial ZNO-1 adsorbent, which exhibited an average ZnO crystal size of 30 nm. The results show that the BET surface area (and pore volume) increased as a function of milling time. The figure also shows that sulfur capture was meaningfully affected by the milling time, with a threefold increase in capacity after 2 h relative to the unmilled sample. The surface area (and pore volume) increase directly contributes to the porosity of the particle, which is essential for the maximum utilization of the reacting solid. Milling contributes to a higher sulfur capture capacity in two ways: (1) in the nanoscale by breaking up crystals for an increased amount of grain boundaries and (2) in the macro scale by improving the porosity, i.e., the available gas–solid contact area. The addition of supporting materials is another method to provide porosity to materials.

#### 3.2.3 Gas Composition

**Figure 6:**
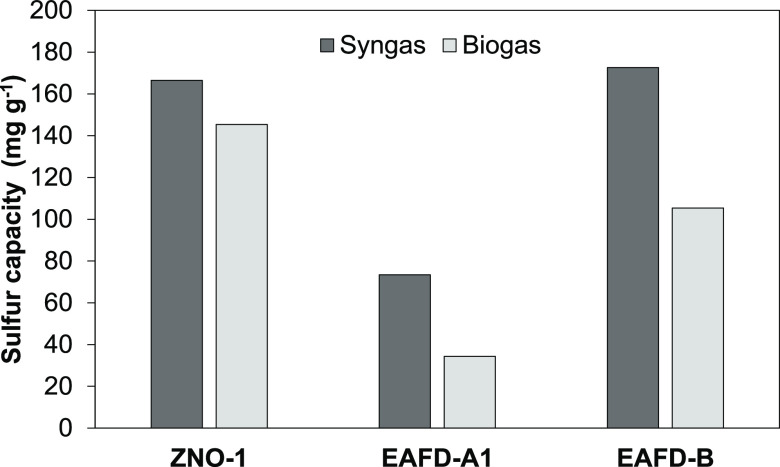
Sulfur capture capacity in syngas and biogas at 400 °C.

The model syngas (H_2_ + CO)/(H_2_O + CO_2_) ratio is 1.34, which is a reducing atmosphere. The model biogas is mainly composed of CH_4_ and CO_2_ and exemplifies a less reducing atmosphere. Figure 6 gives the sulfur capacities at 400 °C in these atmospheres for EAFD-A1, EAFD-B, and ZNO-1.

Figure 6 shows that the sulfur capture capacity was lower in biogas compared to syngas, with EAFD-A1 performance dropping to half of the syngas performance. The reducing atmosphere could improve the availability of the more active ZnO by the reduction of zinc ferrite. The total reaction can be described as
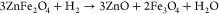
7Zinc ferrite consists of ZnO and Fe_2_O_3_ either as equimolar franklinite or the nonstoichiometric zinc-dislocated franklinite. In reducing atmospheres, it forms ZnO and Fe_3_O_4_ or partly zinc-bearing Fe_3_O_4_, depending on the strength of the reducing atmosphere.^48^ The relative performance improvement of EAFD-A1 in syngas was significantly higher than for EAFD-B. This is consistent with the characterization results, which revealed that for EAFD-A1 approximately 50% of all zinc was in the zinc ferrite form, while for EAFD-B, it was only 15%. Consequently, to maximize the usage of the available active zinc in the EAF dust, a reducing atmosphere is preferred.

#### 3.2.4 Temperature

**Figure 7:**
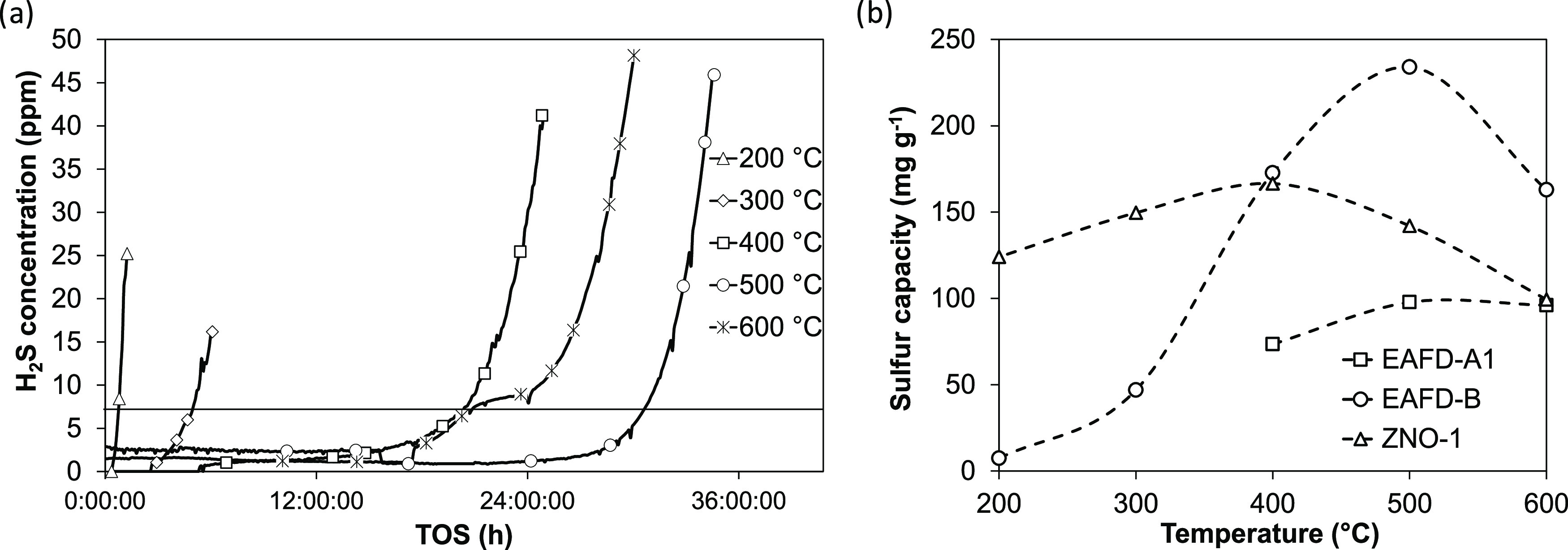
Breakthrough capacity determination in syngas. (a) EAFD-B breakthrough curves. The horizontal line depicts the breakthrough concentration. (b) Sulfur capture capacities as a function of temperature.

The effect of temperatures between 200 and 600 °C on the sulfur capture capacity is presented in Figure 7.

Figure 7a shows that the longest breakthrough time is achieved at 500 °C, while at 200 and 300 °C the sulfur capture capacity was significantly lower than for ZNO-1. This indicates that EAF samples are more temperature-sensitive and better suited for hot desulfurization applications. In contrast to the findings of Su et al.^49^, where EAF samples were tested at 400–700 °C, and the breakthrough time linearly increases with the reaction temperature, the 600 °C EAFD-B breakthrough occurs before 500 °C. EAFD-B achieved a sulfur capture capacity of 234 mg g^–1^ at 500 °C, which constitutes an almost full zinc utilization. Su et al. studied H_2_S adsorption capacities measured in the range of 75–95 mg g^–1^ at conditions with a high H_2_S feed concentration of 10,000 ppm and weight hourly space velocity (WHSV) of 8000 cm^3^ (h × g)^−1^.

**Figure 8:**
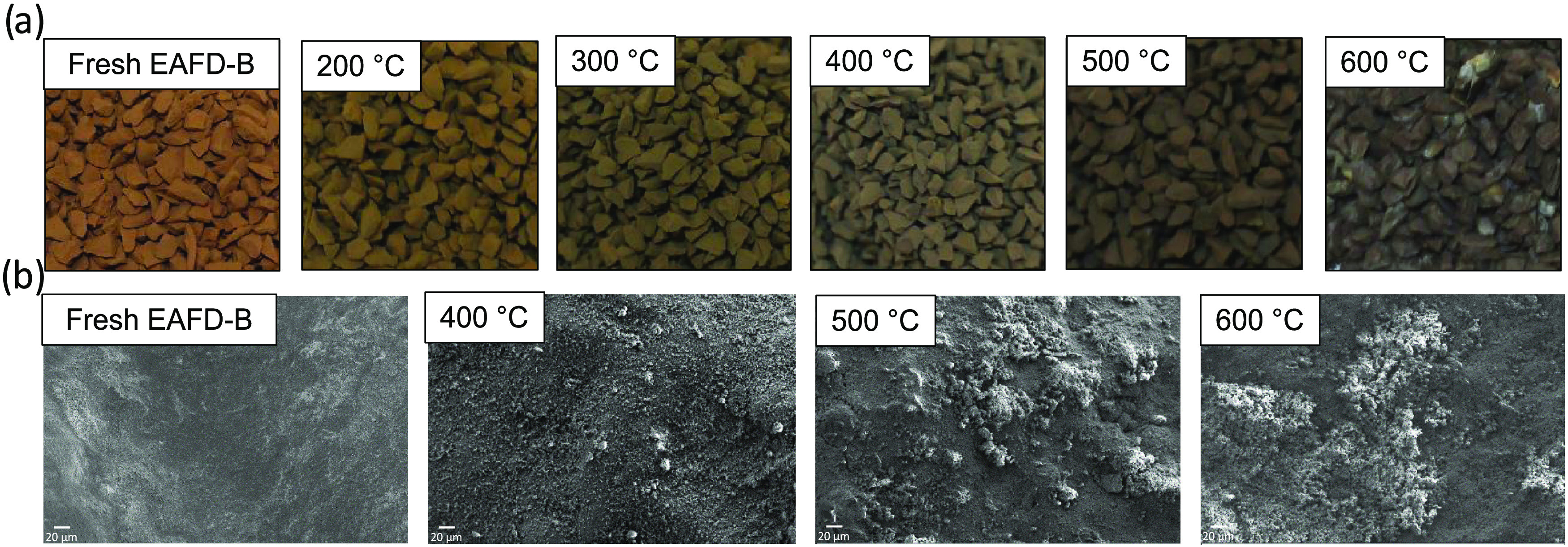
Fresh and spent EAFD-B sample: (a) granulates and (b) granulate SEM images (magnification of 250).

The prebreakthrough minimum residual effluent H_2_S concentration at 500 °C before breakthrough for fresh EAFD-B is 1.5 ppm, and increases at 600 °C to 2.5 ppm. At 400 °C and below, the residual concentration remains below the analytical detection limit. In contrast, a minimum residual H_2_S concentration of ZNO-1 is significantly higher at 4–7 ppm, thus also affecting sulfur capacity, calculated at 7 ppm breakthrough (the 600 °C ZNO-1 sulfur capacity was calculated at 10 ppm breakthrough to compensate for this). Figure 7b shows that also EAFD-A1 performance markedly improves at higher temperatures. However, the absolute capacity is still below EAFD-B. Issues related to zinc volatilization are apparent at 500–600 °C for all of the tested samples. Furthermore, particle fusion issues were detected, especially for EAFD-B. At high conversion rates, the granulates fuse together due to the higher volume of sulfides, exemplifying why full conversion of metal oxides is unattainable in practice^50^. This is a problem, especially for primary metal oxide adsorbents since effectively a part of the valuable material is always left unused. Adding an inert material to the bed or increasing the particle size can alleviate this problem. However, it also artificially limits the effective sulfur capture on a volumetric basis. Figure 8 shows the spent sample visual changes and SEM images.

Figure 8a shows the evolution of the EAFD-B sample color after sulfidation at varying temperatures. The darker colors at higher temperatures give a visual confirmation of greater rates of iron oxide reduction. The SEM images from the spent adsorbent surface, Figure 8b, show the evolution of sulfided areas (light) at increasing temperatures. The EDS area analysis gave sulfur concentrations by mass at 400, 500, and 600 °C of 27.2, 34.4, and 32.1% respectively, which are consistent with the breakthrough results. To further investigate the effect of temperature, the spent 500 °C sample (operated to full breakthrough) characterization was performed, and the results are presented in Table 4.

**Table 4:** Spent 500 °C EAFD-B Analysis Results^a^

	**EAFD-B**
***S*_cap_** (mg g^–1^)	234
**Zinc (% by Mass)**	
Zn as ZnS	41.2 [0.07]
Zn as ZnO	0.6 [41.1]
Zn as zinc ferrite	0.3 [6.9]
Zn°	0.8 [0.06]
**Other analysis**	
carbon (% by mass)	0.4 [0.5]
BET SA (m^2^ g^–1^)	5.5 [16.0]
pore volume (cm^3^ g^–1^)	0.02 [0.09]

a Fresh sample results are indicated in brackets.

The full breakthrough spent sample analysis shows that at 500 °C, over 95% of the total zinc is converted to zinc sulfide. XRD analysis indicated that no FeS was formed. The zinc ferrite reduction is almost complete and the remaining zinc in zinc ferrite amounts to only 0.3% by mass. A similar 600 °C sample characterization reveals that an almost complete reduction of zinc ferrite had also occurred, despite only partial ZnO conversion to ZnS (test was terminated before full breakthrough). According to the analysis results, carbon formation in 500 °C syngas is not an issue. The metallic zinc content in the spent sample is lower than for a 400 °C spent sample, which indicates a slightly higher zinc loss.

High temperatures markedly improve the performance of EAF dust, partly due to improved mass-transfer rates to the active sites and also due to the increased availability of active zinc oxide (from the reduction of zinc ferrite). The characterization results confirm the belief that 500 °C is sufficient to achieve complete zinc ferrite reduction at rates sufficient for maximum utilization of the formed ZnO for sulfur capture. Additionally, the zinc in zinc ferrite is less prone to volatilize, allowing for operation at more demanding conditions, and thus providing an advantage over single-oxide ZnO adsorbents. While iron was shown not to be active, a longer contact time or higher sulfur load in the gas may change this (as indicated by Kobayashi et al.^48^).

The benefits of EAFD, its low cost compared to primary materials, and satisfactory sulfidation performance, make this material an appealing candidate for adsorption applications. The tests showed that although the theoretical sulfur capacity of primary ZnO adsorbents may be higher, full utilization of high Zn-content samples is often challenging in practice, significantly narrowing the performance gap to EAFD adsorbents. Nevertheless, higher zinc-content EAFD materials are strongly preferred, which is evident from the performance difference between the samples, EAFD-A and EAFD-B. This work also showed that EAF dust does not necessarily require heavy processing since merely a milling step was introduced to achieve good adsorbent properties. For industrial applications, however, additional processing is required to, for example, improve adsorbent mechanical properties. On the other hand, additive addition can also alleviate other problems such as COS formation.

## 4 Conclusions

The relative H_2_S breakthrough tests at 400 °C and an SV of 17,000 h^–1^ showed that EAFD-B, with 48% by mass zinc concentration, exhibited a sulfur capture capacity of 170 mg g^–1^, compared to the 168 mg g^–1^ of the reference ZNO-1. The EAFD-A samples displayed lower capture capacities, which were consistent with their lower zinc concentrations. The EAF dust milling time was found to significantly affect the sulfur capture capacity through the increased material porosity and available active surface. A reducing gas atmosphere, such as syngas, was found to increase EAFD sulfur capture capacity. Spent sample characterization indicated that zinc was the only sulfiding species in the test conditions, and zinc from both ZnO and ZnFe_2_O_4_ were active in sulfur capture. The EAFD samples were more temperature-sensitive in terms of sulfidation performance than the reference sample. The relative improvement over ZNO-1 at 500–600 °C was significant, while still retaining acceptable prebreakthrough residual H_2_S concentrations. EAFD-B exhibited the highest capacity of 234 mg g^–1^ at 500 °C, exhausting almost all available zinc before the breakthrough. It was therefore experimentally shown that the high-volume steel manufacturing byproduct, EAF dust, can successfully be applied to hot gas desulfurization applications and even exceed a primary ZnO adsorbent in capture capacity.

## Supporting Information Available

Fresh EAF dust XRD diffractograms; fresh EAF dust trace element analysis results; and thermogravimetry plots of EAF samples (PDF)

## Abbreviations

BET SA: Brunauer–Emmett–Teller surface area
BF–BOF: blast furnace–basic oxygen furnace
DRI: direct reduced iron
EAF: electric arc furnace
EAFD: electric arc furnace dust
ICP-MS: inductively coupled plasma mass spectrometry
ICP-OES: inductively coupled plasma optical emission spectrometry
SEM: scanning electron microscope
TG: thermogravimetry
XRD: X-ray powder diffraction
XRF: X-ray fluorescence
