# Supplementary material for: Steel Manufacturing EAF Dust as a Potential Adsorbent for Hydrogen Sulfide Removal
Source: Energy Fuels. 2022 Mar 17;36(7):3695–703. doi: 10.1021/acs.energyfuels.1c04235 (PMC8996240; doi:10.1021/acs.energyfuels.1c04235)
Supplement: Supplementary file 1 — ef1c04235_si_001.pdf [file ef1c04235_si_001.pdf]

SUPPORTING INFORMATION

Steel manufacturing EAF dusts as potential adsorbents for hydrogen sulfide removal

Christian Frilund[a], Minna Kotilainen[a], José Barros Lorenzo[b], Pertti Lintunen[a], Kimmo Kaunisto[a]*

[a]VTT Technical Research Centre of Finland Ltd., P.O. Box 1000, FI-02044 VTT, Finland

[b]Arcelor-Mittal Global R&D, voie Romaine, Maizières lès Metz, F-57280, France

*Corresponding author. E-mail:christian.frilund@vtt.fi

XRD analysis

Figure S1 shows XRD diffractograms for the fresh EAF dusts.

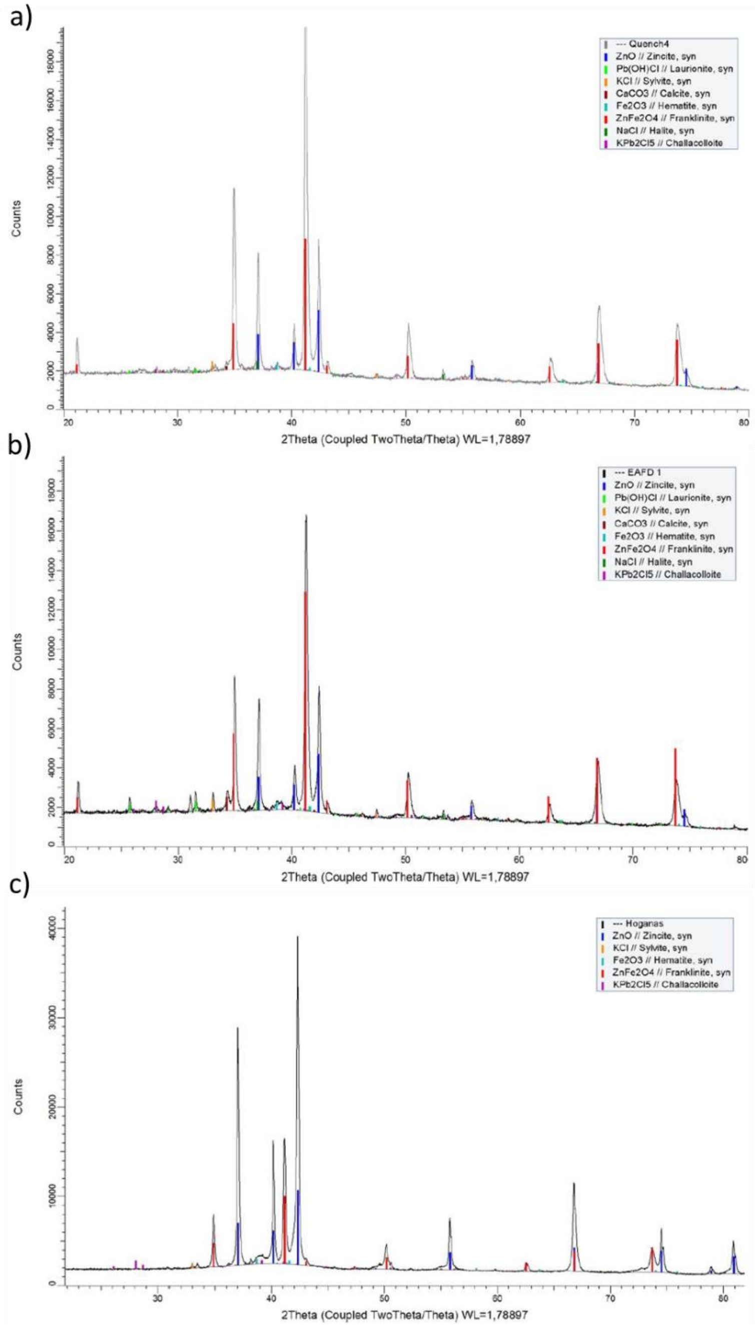

Figure S1. X-ray diffractograms for fresh: a) EAFD-A1. b) EAFD-A2. c) EAFD-B.

Trace element analysis

The trace element analysis results of the EAF dusts are given in

Table **S1**.

Table S1. Characterisation of Fresh EAFD Trace Elements.

	**EAFD**		
	**A1**	**A2**	**B**
**% by mass**			
Cr	0.65	0.63	0.06
Mo	0.01	0.01	0.01
Ni	0.04	0.04	0.01
P	0.15	0.13	0.01
Hf	0.00	0.00	0.00
Sn	0.06	0.07	0.01
Ti	0.07	0.05	0.02
**ppm by mass**			
Ag	39	35	12
As	38	42	11
Bi	111	131	16
Co	32	23	<10
Sb	102	107	12
Se	<10	<10	<10
Te	<10	16	<10
Tl	<10	<10	<10

Thermogravimetry

Figure S2 presents the mass changes as a function of temperature in a N_2 atmosphere.

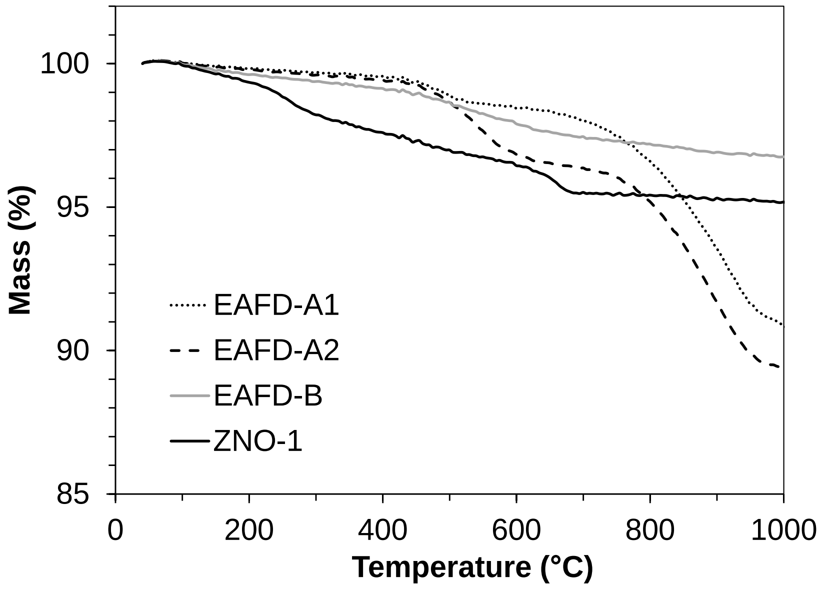

Figure S2. Thermogravimetry plots for fresh samples EAFD-A1, EAFD-A2, EAFD-B, and ZNO-1 in a nitrogen atmosphere.

The weight loss for the EAFD samples is under 2% by mass up to 500 °C, while for the reference ZNO-1, the weight loss amounted to 3%. Two distinct weight loss regions are recognized, 500−550 °C, which could be attributed to metallic zinc volatilization, and 800−900 °C for EAFD-A1, which exceeds the ZNO-1 weight loss, indicating non-zinc species volatilization.
